# All-Covalent Organic Framework Nanofilms Assembled Lithium-Ion Capacitor to Solve the Imbalanced Charge Storage Kinetics

**DOI:** 10.1007/s40820-024-01343-2

**Published:** 2024-02-15

**Authors:** Xiaoyang Xu, Jia Zhang, Zihao Zhang, Guandan Lu, Wei Cao, Ning Wang, Yunmeng Xia, Qingliang Feng, Shanlin Qiao

**Affiliations:** 1 College of Chemistry and Pharmaceutical Engineering, Hebei University of Science and Technology, Shijiazhuang, 050018 People’s Republic of China; 2 School of Chemistry and Chemical Engineering, Northwestern Polytechnical University, Xi’an, 710072 People’s Republic of China; 3 Hebei Engineering Research Center of Organic Solid Photoelectric Materials for Electronic Information, Shijiazhuang, 050018 People’s Republic of China

**Keywords:** Covalent organic frameworks, Lithium-ion capacitor, Charge storage kinetic

## Abstract

Free-standing covalent organic framework (COFs) nanofilms exhibit a remarkable ability to rapidly intercalate/de-intercalate Li^+^ in lithium-ion batteries, while simultaneously exposing affluent active sites in supercapacitors. The development of these nanofilms offers a promising solution to address the persistent challenge of imbalanced charge storage kinetics between battery-type anode and capacitor-type cathode in lithium-ion capacitors (LICs). Herein, for the first time, custom-made COF_BTMB-TP_ and COF_TAPB-BPY_ nanofilms are synthesized as the anode and cathode, respectively, for an all-COF nanofilm-structured LIC. The COF_BTMB-TP_ nanofilm with strong electronegative–CF_3_ groups enables tuning the partial electron cloud density for Li^+^ migration to ensure the rapid anode kinetic process. The thickness-regulated cathodic COF_TAPB-BPY_ nanofilm can fit the anodic COF nanofilm in the capacity. Due to the aligned 1D channel, 2D aromatic skeleton and accessible active sites of COF nanofilms, the whole COF_TAPB-BPY_//COF_BTMB-TP_ LIC demonstrates a high energy density of 318 mWh cm^−3^ at a high-power density of 6 W cm^−3^, excellent rate capability, good cycle stability with the capacity retention rate of 77% after 5000-cycle. The COF_TAPB-BPY_//COF_BTMB-TP_ LIC represents a new benchmark for currently reported film-type LICs and even film-type supercapacitors. After being comprehensively explored via ex situ XPS, ^7^Li solid-state NMR analyses, and DFT calculation, it is found that the COF_BTMB-TP_ nanofilm facilitates the reversible conversion of semi-ionic to ionic C–F bonds during lithium storage. COF_BTMB-TP_ exhibits a strong interaction with Li^+^ due to the C–F, C=O, and C–N bonds, facilitating Li^+^ desolation and absorption from the electrolyte. This work addresses the challenge of imbalanced charge storage kinetics and capacity between the anode and cathode and also pave the way for future miniaturized and wearable LIC devices.

**Supplementary Information:**

The online version contains supplementary material available at 10.1007/s40820-024-01343-2.

## Highlights

- An all-covalent organic framework (COF) nanofilm-structured lithium-ion capacitor (LIC) was developed by custom-made COF nanofilms as the anode/cathode.
- The COF nanofilm-structured LIC exhibits good electrochemical properties via the fast Li^+^ transport kinetics of the anodic COF_BTMB-TP_ nanofilm and the high specific capacity of the cathodic COF_TAPB-BPY_ nanofilm.
- This work can realize the charge storage kinetics and capacity balance of anode/cathode in COF_TAPB-BPY_//COF_BTMB-TP_ LIC.

**Supplementary Information:**

The online version contains supplementary material available at 10.1007/s40820-024-01343-2.

## Graphical abstract

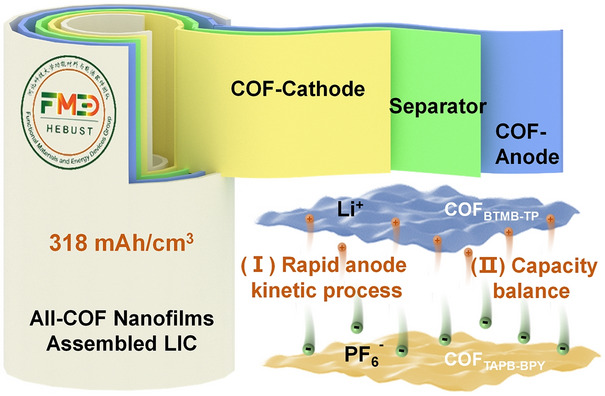

## Introduction

Lithium-ion capacitors (LICs) integrate the lithium-ion battery-type anode and capacitor-type cathode into one configuration in the lithium-salt-dissolving organic electrolyte, bridging the gap of two energy storage devices in terms of energy/power density and cycle lifetime [1]. From a mechanical perspective, LICs display a distinctive and simultaneous asymmetrical charge storage process. Within these energy storage devices, lithium metal ions intercalate and de-intercalate within the anode, while anions adsorb and desorb at the cathode. As a result, two electrodes operate with discrete mechanisms across different potential ranges, contributing to the unique performance characteristics of LICs. In most reports of LICs, high energy density is typically achievable only at low currents, with a rapid decrease observed at higher currents. The primary issue lies in the imbalanced charge storage kinetics between the two electrodes. The Li^+^ intercalation/de-intercalation process at the battery-type anode is more sluggish compared to the electrical double-layer upon polarization at the capacitor-type cathode/electrolyte interface [2, 3]. Thus, achieving simultaneously competitive energy/power density, along with excellent rate capability, poses an extreme challenge involving in the entire LIC system.

At present, a wide array of active materials, encompassing transition metal oxides, sulfides, nitrides, Li_4_Ti_5_O_12_ (LTO), Li_3_VO_4_, TiO_2_, and carbon-based substances [4–7], have emerged as promising contenders for anode materials in LICs. Notably, recent refreshing progress have been made in LICs mainly assembled with lithium-intercalation compounds and activated carbon (AC) as cathode and anode, respectively [8]. However, LICs constructed with insertion-type materials encounter inherent challenges, especially when serving as battery-type anodes in LICs, which rely on alloying/dealloying and conversion reactions. These challenges include intrinsic poor electronic conductivity, low theoretical capacities, sluggish kinetics, and significant volume variation upon lithiation/de-lithiation [9], resulting in reduced energy and power densities, as well as compromised cycling stability. Thus, addressing this challenge and achieving enhanced electrochemical performance in LICs necessitates the development of innovative configurations for both the anode and cathode.

Considering the notable advantages over inorganic materials in the realm of LICs, such as their high specific surface area, versatile structural adaptability, extended *π*-conjugated structures, and abundant redox-active sites, porous organic polymers (POPs) emerge as another highly appealing class of electrode materials for LICs [10]. Two-dimensional layered covalent organic frameworks (2D COFs), as a crystalline class of POPs, offer some distinct advantages in the Li^+^ insertion kinetics, mainly containing the high ionic transport and electrical conductivity from their hereafter-mentioned peculiarities: (i) 2D COFs stack functional *π*-electron systems in *Vander Waals* contact, attaching maximal *π*-orbital overlap for charge transport, and also exhibit open porous channels parallel to the stacking direction [11]. (ii) Well-defined and predictable nanopores can allow the rapid ion/charge diffusion to reach the build-in active sites and simultaneously hold the electrolyte ions [12]. (iii) The diversity of building blocks, coupled with atomically controllable preparation, facilitates the precise integration of redox-active groups, allowing for the customization of Faradaic redox properties within COFs [13]. COFs have been investigated as promising electrode materials for supercapacitors and lithium-ion batteries [14]. To the best of our knowledge, academic investigations concerning the application of bulk COFs as anode materials for LICs are still in their nascent stages. This may be mainly due to the lack of thoroughly mechanism about COF anode Li^+^ intercalation/de-intercalation, suitable capacitor-type cathodes along with the complicated device assembly process in nonaqueous LICs. In addition, the robust *π*‒*π* stacking interactions between layers of COFs pose a constraint on the efficient transport of lithium-ions to the active sites situated within the bulk COFs. This suboptimal utilization of active sites presents a formidable obstacle for COFs in meeting the demanding criteria for high-capacity output and exceptional rate capability, essential for LIC anodes. It is worth noting that recent progresses have mainly focused on strengthening the electronic conductivity and lithium-ion accessibility of battery-type anode active materials, though the design/synthesis of novel high-rate anode nanomaterials, or hybridization with electrically conductive substrates [15–17]. Nevertheless, less attention has been paid to the ordered microstructure configuration design of whole electrode, especially for overall electrode system with fast mass and free electron transfer.

In fact, there is a ceiling placed on achievable charge storage kinetics that is locked by the inevitable agglomeration/stacking of conventional electrodes. An ideal electrode configuration should effectively utilize active material domains and optimize electron/ion transport pathways throughout the electrode, without altering material chemistry [18]. In our previous research, we discovered that free-standing COF nanofilms possess exceptional capacitive performance when utilized as micro-supercapacitor interdigital electrodes [19], which can be expected as the promising practical application based on the reports [20–23]. Therefore, free-standing COF nanofilms have the ability to achieve the high structural integrity and interconnectivity throughout the whole battery-type anode. This is made possible by the 2D conjugated skeleton, which allows for free electron conduction and *π*‒π* electron transition in 3D interlayer. Additionally, 1D porous channels facilitate fast electrolyte ion transport in a longitudinal and parallel interlayer without obvious “dead volume”. However, the areal mass of COF nanofilm anode is far below that of commonly used bulk AC cathode, leading to the mass imbalance between two electrodes that enormously impacts the LIC energy density.

Herein, we propose a molecule-level structural design strategy for integrated design-construction of all-COF nanofilms, which can be used in the assembly of LICs. This involves the polymerization of COF_BTMB-TP_ nanofilm with 2,2’-bis(trifluoromethyl)benzidine (BTMB) and 2,4,6-Triformylphloroglucinol (TP) as anode, and COF_TAPB-BPY_ nanofilm, utilizing 1,3,5-tris(4-aminophenyl)benzene (TAPB) and 2,2'-bipyridyl-5,5'-dialdehyde (BPY) as cathode. The designed concepts are as follows: (i) COF_BTMB-TP_ nanofilm with strong electronegative–CF_3_ groups can adjust the partial electron cloud density for Li^+^ migration to ensure the rapid anode kinetic process, releasing much Li^+^ on account of F competitive advantage over PF_6_^−^ electrolyte. COF_BTMB-TP_ nanofilm provides abundant Li^+^ storage sites from the highly reversible semi-ionic/ionic state of C–F bonds. (ii) The demonstrated capacitance-dominated charge storage processes in the COF_BTMB-TP_ nanofilm anode permit fast kinetics for the transported lithium-ions and electrons, ensuring well-matching kinetics between cathode and anode, and contributing to outstanding performance. (iii) Porous COF_TAPB-BPY_ film cathode with inherent skeleton nitrogen atoms not only provide additional charge storage sites but also match with COF_BTMB-TP_ film anode in the areal mass with the same order of magnitude. The density functional theory (DFT) calculations were performed to identify Li^+^ insertion sites and diffusion paths for COF_BTMB-TP_ nanofilm anode. In terms of mismatch capacity from cathode/anode with different energy storage modes, the thickness of COF_TAPB-BPY_ nanofilm cathode was optimized to fit with the COF_BTMB-TP_ anode in the capacity. Then, the assembled COF_TAPB-BPY_//COF_BTMB-TP_ all-COF nanofilm LIC is successfully fabricated and optimized in both kinetic and mass balance, demonstrating the high energy density of 318 mWh cm^−3^ at a power density of 6 W cm^−3^, long cycle stability with the capacity retention rate of 80% after 1000-cycle, and good rate capability.

## Experimental

### Materials

The sodium dodecylbenzene sulfonate (SDBS, 98%) and TAPB (99.67%) were purchased from Tianjin Damao Chemical Reagent Factory, while BTMB (99.98%) and TP (97%) were obtained from Jilin Province Yanshen Technology Co, Ltd. BPY (97%) and glacial acetic acid (99.5%) were procured from Shanghai Macklin Biochemical Co, Ltd. Electrolytes (1 M LiPF_6_) in ethylene carbonate (EC)/dimethyl carbonate (DMC)/ethyl methyl carbonate (EMC) (volume ratio of 1:1:1) and CR2032 coin type cells were purchased from DoDochem.

### Preparation of COF_TAPB__-BPY_ and COF_BTMB__-TP_ Nanofilms

The SDBS (1 mg mL^−1^ in chloroform solvent, 20 μL) was dripped into the deionized water (50 mL) in crystal dish, to be dispersed on the liquid surface for 40 min. The TAPB monomer (1 mg mL^−1^ in 0.12 M HCl solution) was added into the liquid phase under SDBS and maintained for 1 h. And then another BPY monomer (1 mg mL^−1^ in 0.12 M HCl solution) and acetic acid catalyst were added to complete the polymerization reaction of TAPB and BPY monomers. After 7 days, the COF_TAPB-BPY_ nanofilm was grown between the gas/liquid interface [24–26].

Similarly, the COF_BTMB-TP_ film was synthesized by the same method, except for the TP (2 mg mL^−1^ in 0.12 M HCl solution) and BTMB (2 mg mL^−1^ in 0.12 M HCl solution) monomers, without additional acetic catalyst.

### Materials Characterizations

The chemical structures of COF_TAPB-BPY_ and COF_BTMB-TP_ nanofilms were investigated by Fourier transform infrared spectroscopy (FTIR, *Thermo Scientific Nicolet iS10 spectrometer*), Raman (*Renishaw qontor*), X-ray photoelectron spectroscopy (XPS, *Thermo Scientific K-Alpha spectrometer*) test. The morphologies of COF_TAPB-BPY_ and COF_BTMB-TP_ nanofilms were observed by the optical microscope (OM, *LEICA DM750M*), scanning electron microscope (SEM, *JEOL*), transmission electron microscopy (TEM, *D/MAX-2500, Rigaku*) and atomic force microscope (AFM, *Dimension Icon, Bruker*). The surface morphologies of COF_TAPB-BPY_ and COF_BTMB-TP_ nanofilms were monitored by the scanning electrochemical microscopy (SECM, *VersaSCAN, Advance Measurement Technology, InC*) though a three-electrode system including COF nanofilms coated Si wafer, Ag/AgCl reference and probe in K_3_Fe(CN)_6_/K_4_Fe(CN)_6_ electrolyte (0.1 mmol L^−1^). The lipophilicity of COF_BTMB-TP_ was tested by ^7^Li solid-state NMR (*Agilent 600M*) analysis, after soaking COF_BTMB-TP_ nanofilm in LiPF_6_ solution.

### Half-Cell and Full-Cell Assembly

The prepared COF_BTMB-TP_ anode nanofilm was transferred to a copper foil (*ϕ* = 12 mm) to assemble into the half-cell device, with polypropylene film and Li metal as the separator and reference electrode, respectively, using the 1 M LiPF_6_ in EC/DME/EMC (volume ratio, 1:1:1) (40 μL) as the electrolyte, in the CR2032 coin cell. All the assembly process was carried out in the argon-filled glove box (*Etelux,* water and oxygen content of less than 0.1 ppm).

Then, the COF_BTMB-TP_ half-cell was treated by charge/discharge cycles at 0.1 C in the voltage range of 0‒2 V versus Li/Li^+^ on LAND battery testing system to achieve pre-lithiation of COF_BTMB-TP_ nanofilm anode. After pre-lithiation, the COF_BTMB-TP_ half-cell was transferred into the glove box and remove the negative tab. The obtained pre-lithiated COF_BTMB-TP_ anode nanofilm was assembled into the COF_TAPB-BPY_//COF_BTMB-TP_ LIC, with COF_TAPB-BPY_ nanofilms as the cathode, in the similar process with above full-cell assembly.

### Materials Electrochemical Characterizations

Electrochemical tests contain the cyclic voltammetry (CV), galvanostatic charge/discharge (GCD), electrochemical impedance spectroscopy (EIS), which can be performed in the Princeton Applied Research electrochemical workstation (*VersaSTAT 3*), except GCD in the LAND battery testing system.

CV measurement of COF_TAPB-BPY_//COF_BTMB-TP_ LIC was performed in the potential range of 0‒2 V at various scan rates from 10 to 100 mV, and GCD test with the voltage window of 0‒1.5 V at different current densities from 0.01 to 0.1 mV cm^−2^. In these CV curves, the current can be divided into surface capacitance (*k*_1_*v*) and diffusion-control reaction (*k*_2_*v*^1/2^), which can be further quantified as Eq. (1) [27]:

1 $$i={k}_{1}v+{k}_{2}{v}^{1/2}$$

In order to assess the charge storage capacity of these COF_TAPB-BPY_//COF_BTMB-TP_ LIC devices, the important index such as areal specific capacitance (*C*_A_, mF cm^−2^), volumetric specific capacitance (*C*_v_, mF cm^−3^), energy density (*E*, Wh cm^−3^) and power density (*P*, W cm^−3^) can be calculated based on the GCD curves, which can be derived from Eqs. (2‒5) [28, 29]:

2 $${C}_{A}=\frac{i}{\times} {\Delta t}{\Delta v\times A}$$

3 $${C}_{V}=\frac{i\times \Delta t}{\Delta v\times A\times h}$$

4 $$E=\frac{1}{2}{\times C}_{V}\times \frac{{\left(\Delta V\right)}^{2}}{3600}$$

5 $$P=\frac{E}{\Delta t}\times 3600$$

where *i* (A) is the discharge current, Δ*t* (s) is the discharge time, Δ*v* (V) is the potential windows, A (cm^2^) and *h* (cm) are the area and thickness of active material.

EIS plots of COF_TAPB-BPY_//COF_BTMB-TP_ LIC device was tested in the frequency range of 10 mHz to 100 kHz with an applied potential amplitude of 5 mV. In addition, the EIS plots of anode COF_BTMB-TP_ nanofilm and bulk COF_BTMB-TP_ were also measured at different voltages ranging from 0.01 to 2 V versus Li/Li^+^, to determine the *Warburg* impedance (*W*_*s*_), which is closely related with the Li^+^ diffusion coefficient (*D*'_Li_^+^, cm^3^ s^−1^), as shown in Eq. (6) [30]:

6 $${D}_{{{\text{Li}}}^{+}}{\prime}=\frac{{R}^{2}{T}^{2}}{2h{A}^{2}{n}^{4}{F}^{4}{C}^{2}{\sigma }_{\omega }^{2}}$$

where *n* is the number of the electrons involved in the electrode reaction, *σ*_*ω*_ value is the slope of Z ~ *ω*^1/2^ plots, *C* (mol cm^−3^) is the concentration of lithium-ions in the lattice (roughly estimated as electrolyte concentration).

## Results and Discussion

### COF Nanofilm Preparation and Characterizations

**Scheme 1:**
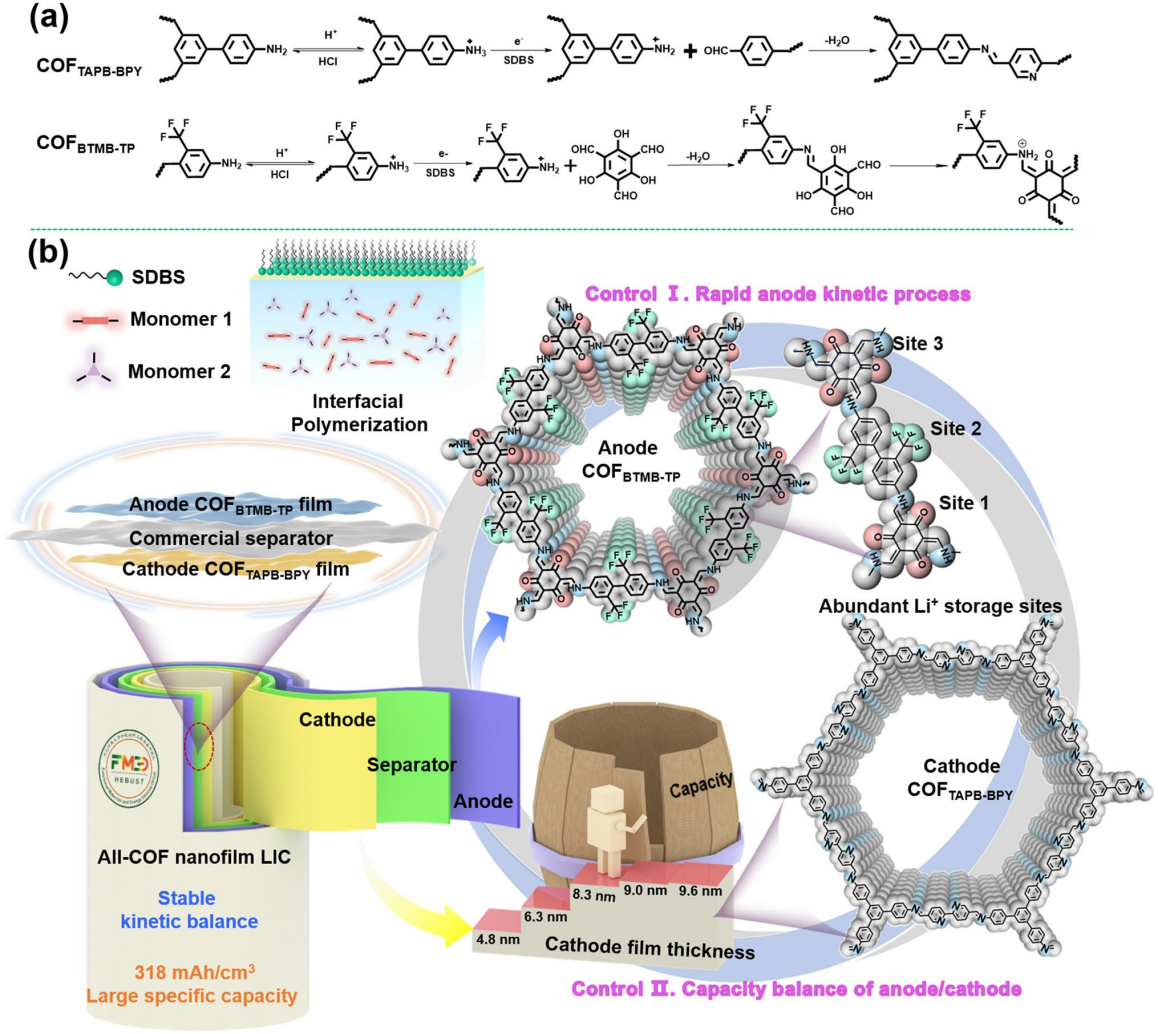
Schematic synthesis of COF_TAPB-BPY_ and COF_BTMB-TP_ nanofilms (**a**); illustration of assembling COF_TAPB-BPY_//COF_BTMB-TP_ nanofilm LIC (**b**)

Scheme 1 illustrates the synthesis of COF_TAPB-BPY_ and COF_BTMB-TP_ nanofilms, which are then assembled into COF_TAPB-BPY_//COF_BTMB-TP_ nanofilm LIC. The COF nanofilms were prepared through the reversible Schiff base polymerization of BTMB and TP monomers (or TAPB and BPY) using a soft template derived the surfactant SDBS. The COF_BTMB-TP_ nanofilm was generated by undergoing the irreversible *enol‒to‒keto* tautomerization after above reversible Schiff base reaction, which only involves the chemical bond conversion, without destroying any crystallinity [31]. Considering the possible rapid energy storage kinetic process of COF_BTMB-TP_ nanofilm with strong electronegativity–CF_3_ groups, the COF_BTMB-TP_ nanofilm acted as the LIC anode, to match with the capacitance-type cathode. The COF_TAPB-BPY_ nanofilm was applied as the LIC cathode, whose thickness was optimized by controlling the concentration of adding monomers, to obtain the high-performance COF_TAPB-BPY_//COF_BTMB-TP_ LIC device.

**Fig. 1:**
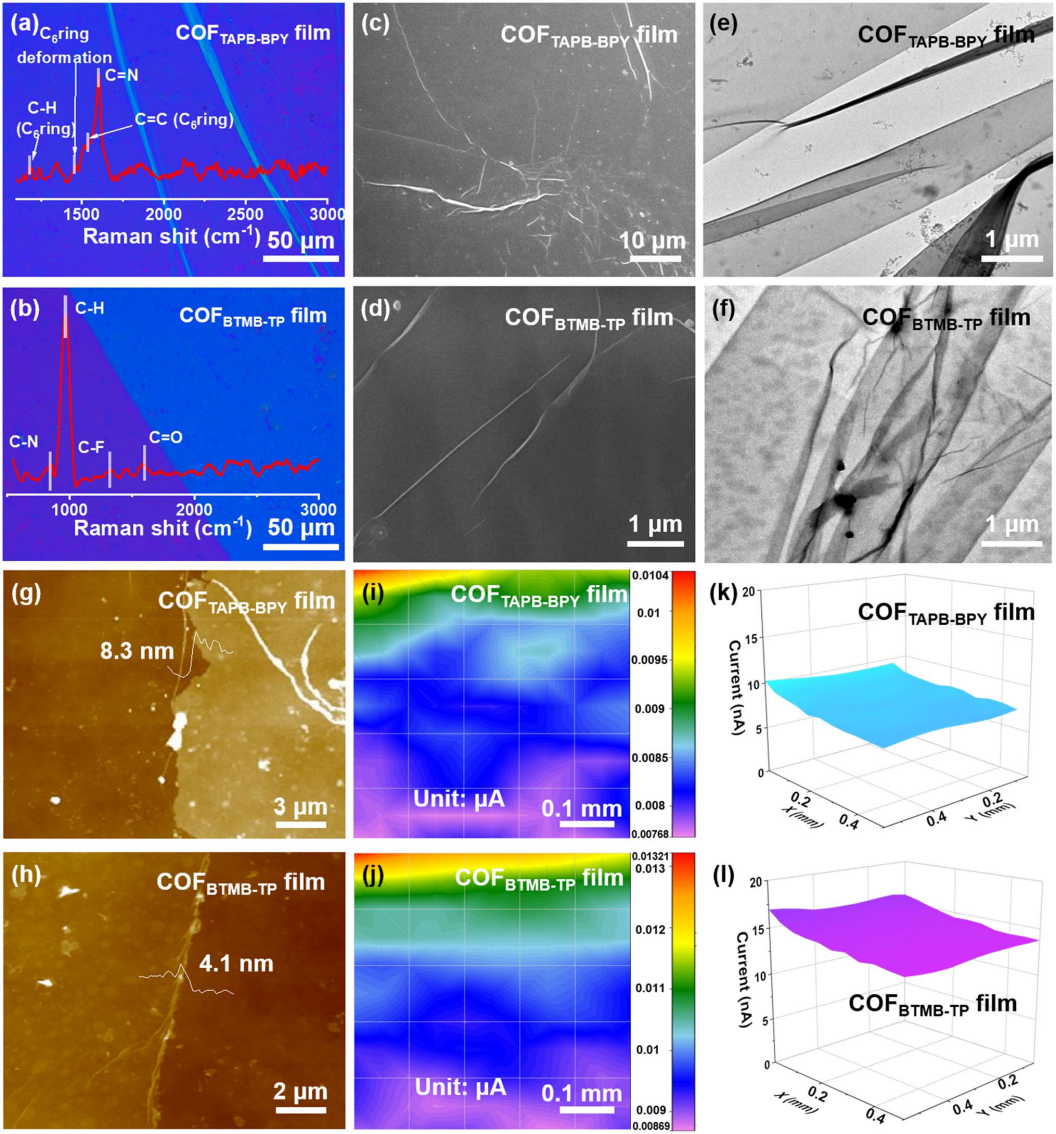
OM (**a‒b)**, Raman spectra (inset)), SEM (**c‒d**), TEM (**e‒f**), AFM (**g‒h**), SECM (**i‒l**) images of COF_TAPB-BPY_ and COF_BTMB-TP_ nanofilms

The chemical structures of COF_TAPB-BPY_ and COF_BTMB-TP_ nanofilms were demonstrated by FTIR, Raman and XPS techniques. As shown in Fig. S1a, the C=N stretching and bending vibration peaks newly appear in 1623 and 1252 cm^−1^, respectively. Additionally, the N–H stretching vibration peak at 3218 cm^−1^ in TAPB and the–CHO stretching vibration peak at 1630 cm^−1^ in BPY disappear. These changes can be attributed to the Schiff base reaction between the TAPB and BPY, resulting in the formation of the C=N bond in the COF_TAPB-BPY_ film [32]. However, the COF_BTMB-TP_ (Fig. S1b) contains newly generated C=O at 1631 cm^−1^ and C–N at 1188 cm^−1^ stretching vibration peaks, confirming the occurrence of *enol-to-keto* tautomerism and the accurate synthesis of COF_BTMB-TP_ film [33], where the C–F bonds at 1049 and 1009 cm^−1^ are not involved in the reaction. Meanwhile, the C=N bond at 1602 cm^−1^, aromatic C–H/C=C vibration bond severally at 1181 and 1542 cm^−1^, deformation vibration of benzene ring at 1460 cm^−1^ [34], can be detected in the COF_TAPB-BPY_ Raman spectrum (inset of Fig. 1a). This provides an initial indication of the successful polymerization between TAPB and BPY. On the other hand, in the Raman spectrum of COF_BTMB-TP_ (inset of Fig. 1b) without the presence of–NH_2_,–CHO, or–OH groups, different vibrational bonds are detected: C–N at 846 cm^−1^, C–H at 965 cm^−1^, C–F at 1317 cm^−1^, C=O at 1594 cm^−1^, and deformation vibration of the benzene ring at 1455 cm^−1^. These observations further confirm the Schiff base reaction between the BTMB and TP monomers. It is worth noting that in the case of COF_BTMB-TP_, there is also evidence of *enol‒to‒keto* tautomerization, leading to the formation of C=C–N bonds.

Figure S1c shows the P-XRD patterns of bulk COF_BTMB-TP_ and COF_TAPB-BPY_, which can be used to reflect the COF_BTMB-TP_ and COF_TAPB-BPY_ nanofilm. The diffraction peak of (100) crystal plane appear at 2*θ* = 2.9° for COF_BTMB-TP_ and 2*θ* = 2.2° for COF_TAPB-BPY_, which can be further calculate the pore size of COF_BTMB-TP_ about 2.9 nm and COF_TAPB-BPY_ about 3.9 nm. Moreover, the structural result of COF_BTMB-TP_ and COF_TAPB-BPY_ can be also confirmed by simulation in Fig. S1d. The COF_BTMB-TP_ by A–A stacking arrangement exhibits the pore size of 2.9 nm, and layer spacing of 0.44 nm. The COF_TAPB-BPY_ by A–A stacking arrangement exhibits the pore size of 3.9 nm, and layer spacing of 0.35 nm. XPS analysis was performed to further research the chemical elements/bonds of COF_TAPB-BPY_ and COF_BTMB-TP_. The C 1*s* core-level spectrum (Fig. S2a) of COF_TAPB-BPY_ can be divided into C−C/C=C at 284.2 eV, C=N in imine group at 284.5 eV [35], while C−F at 292 eV, C=O at 287.9 eV, C−N at 284.5 eV, C−C/C=C at 283.9 eV for COF_BTMB-TP_ [36], further proving the polymerization of TAPB-BPY, coupling of enol‒to‒keto tautomerization of BTMB-TP monomer pair. The N 1s spectrum (Fig. S2b) contains the C−N at 399.6 eV and C−N at 398.4 eV for COF_TAPB-BPY_, while only C−N peak at 399.5 eV for COF_BTMB-TP_. In addition, the unique O and F elements for COF_BTMB-TP_ were also analyzed as follows: C=O bond at 533.5 eV (Fig. S2c) in the O 1*s* spectrum, the C−F bond at 688.25 eV in F 1*s* spectrum, which show that the C−F bond exists in the form of semi-ionic bond in COF_BTMB-TP_ nanofilm (Fig. S2d). 

The OM, SEM, TEM and AFM images of COF nanofilms were collected to observe their morphology. As shown in Fig. 1a, b, the OM photographs show that the smooth and uniform nanofilms can be distinguished for COF_TAPB-BPY_ and COF_BTMB-TP_ nanofilms, without obvious cracks and defects, even if the SEM (Fig. 1c, d) and TEM (Fig. 1e, f) images at a high magnification. The recognized slight folds and edges are the typical of large-area 2D nanofilm. Notably, these COF nanofilms can be transferred into different substrates without destroying the integrity of nanofilms, indicating the high mechanical strength. The thickness of COF_TAPB-BPY_ and COF_BTMB-TP_ nanofilms is measured to be about 8.3 and 4.1 nm, respectively, based on AFM test (Fig. 1g, h). In the 2D and 3D SCEM images (Fig. 1i–l), the observed maximum current signal difference resulting from the redox reaction within the 0.5 × 0.5 mm^2^ scanning region is about 2.5 nA. It is indicated the presence of a flat and intact film across a large surface area for the prepared COF_TAPB-BPY_ and COF_BTMB-TP_ nanofilms, as determined by monitoring the distance between the nanofilm surface and probe.

### Electrochemical Performance of COF_BTMB__-TP_ Nanofilm Electrode

In view of the absorption of special COF on Li^+^, the lithium storage performance of COF_BTMB-TP_ nanofilm was evaluated in a half-cell with lithium metal as the counter electrode. In Fig. S4, a pair of distinct oxidation/reduction peaks separately at 1.3 and 0.73 V can be detected in the CV curves of 0‒2 V* versus *Li/Li^+^ at scan rate of 0.2 mV s^−1^, corresponding to the fact as follows: (i) the F^‒^ and Li^+^ can be bonded by the electrostatic attraction, in virtue of the C–F transformation from semi-ionic into ionic bond (charge). (ii) Afterward, the F semi-ionic bond to nearby carbocation will be reformed, to attach the Li^+^ de-intercalation (discharge). As reported, the highly reversible conversion of C–F ionic/semi-ionic bond exists in the charge/discharge process of C–F contained 2D conductive materials [37], which can provide enormous lithium storage active sites and improve the cycle stability. In addition, there is no obvious variation for CV integral area in the first three cycles, proving that the high reversibility of Li^+^ intercalation/de-intercalation.

**Fig. 2:**
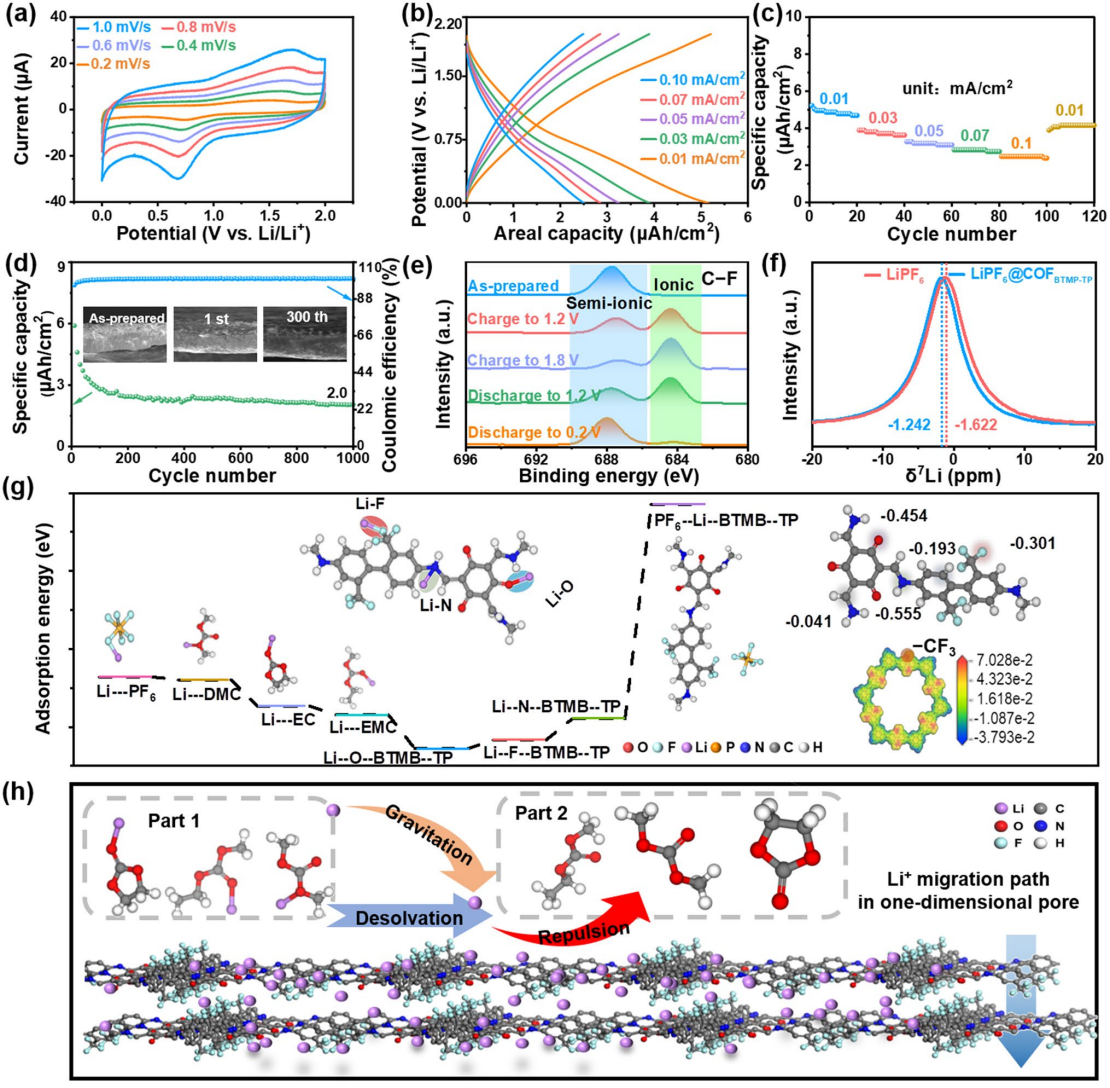
CV curves at different scan rates (**a**), GCD curves at different current densities (**b**), rate capacity (**c**), cycling stability properties after 1000 cycles (**d**) and ex situ XPS spectra of C–F bonds in LiClO_4_ (1 M, in EC:PC = 1:1 Vol%) alternative electrolyte (**e**) for COF_BTMB-TP_ electrode; solid-state ^7^Li NMR spectra of LiPF_6_ with and without COF_BTMB-TP_ addition (**f**); DFT simulated Li^+^ adsorption energy about COF_BTMB-TP_ and electrolyte components (**g**), calculated electrostatic potential distribution of COF_BTMB-TP_ (inset, **g**); schematic descriptions of Li.^+^ migration process from electrolyte components to COF_BTMB-TP_ (**h**)

The similar-shaped CV curves at different scan rates (Fig. 2a) and the GCD curves at different current densities (Fig. 2b) are also the signal of stable electrochemical performances of COF_BTMB-TP_ electrode. Based on the discharge time in GCD curves, the high areal specific capacity of COF_BTMB-TP_ electrode is 5.15, 3.90, 3.25, 2.86 and 2.50 μAh cm^−2^ at current densities of 0.01, 0.03, 0.05, 0.07 and 0.1 mA cm^−2^, respectively. The COF_BTMB-TP_ electrode with only the thickness of 4.1 nm exhibits the extremely high volumetric specific capacity, contrasting significantly with currently reported commercial LiCoO_2_ (LCO)/Li_4_Ti_5_O_12_ electrode with the thickness of 1300 μm and an area specific capacity of 28.6 mAh cm^−2^ [38]. This proves the excellent lithium storage capacity of COF_BTMB-TP_ nanofilm. The high specific capacity of COF_BTMB-TP_ electrode benefits from the unique highly ordered 1D channel, which enables full exposure of Li^+^ to active sites, along with the presence of abundant active sites such as C–F, C=O, C–N. Moreover, because of the thickness of COF_BTMB-TP_ film at nanometer level, it can overcome the problem of covering the active site by layer accumulation of powder COF material. So that all lithium storage sites can be fully utilized. In Fig. 2c, it is observed that the specific capacity retention maintains about 50% even with an increased current density from 0.01 to 0.1 mA cm^−2^*,* and it still approaches to 90% when the current density cycles back to 0.01 mA cm^−2^. The good rate capability of COF_BTMB-TP_ is an unattainable target especially for organic compounds. The COF_BTMB-TP_ electrode shows acceptable cycle performance after 1000 cycles with a specific capacity of 2.0 μAh cm^−2^ at 0.01 mA cm^−2^, and coulombic efficiency with no obvious deformation (Fig. 2d). This performance can be explained by the similar morphology in SEM images obtained after 300th with 1st cycle (inset of Fig. 2d). Compared to the original nanofilm, the obvious solid electrolyte interface (SEI) layer can be found in the cross section of nanofilm after 1st charge/discharge cycle, which exhibit no obvious variation after the following 300 cycles. This good rate capability and structural stability can be attributed to the enhanced structure and SEI stability, which result from the covalent-bonded rigid skeleton structure and C–F bonds.

In the *ex situ* XPS spectra of COF_BTMB-TP_ nanofilm in LiClO_4_ (1 M, in EC:PC = 1:1 Vol%) alternative electrolyte (Fig. 2e), all semi-ionic C‒F bonds exist in the original state, gradually transitioning into ionic C‒F bonds on account of Li^+^ addition during charging, and further recover to semi-ionic C‒F bonds due to the Li^+^ removal during the discharge process. This realizes the highly reversible conversion of semi-ionic to ionic C‒F bonds throughout the whole lithium storage process. Similarly, in Fig. S4b, c,  the lithium storage mechanism of C=O and C‒N bonds was also confirmed in the charge/discharge process. In the ^7^Li solid-state NMR spectra, the chemical shift of Li^+^ transforms from −1.622 to −1.242 ppm after adding COF_BTMB-TP_ nanofilm (Fig. 2f). This shift is attributed to the decreasing local electron cloud density caused by the presence of strong electron-withdrawing–CF_3_ and C=O groups, confirming the interaction between COF_BTMB-TP_ and LiPF_6_. In order to verify these Li^+^ storage active sites at the theoretical level, the corresponding lithium absorption energy values were calculated using *DMol3* module based on DFT. In Fig. 2g, the Li^+^ absorption energy values for oxygen (‒1.37 eV), nitrogen (‒1.13 eV) and fluorine (‒1.31 eV) atoms in COF_BTMB-TP_ are higher than those of PF_6_^−^ (‒0.82 eV), EC (‒0.84 eV), DMC (‒1.04 eV) and EMC (‒1.11 eV) in electrolyte. This observation indicates the strong interaction between COF_BTMB-TP_ and Li^+^, making it more favorable for Li^+^ to bind with COF after separation from electrolyte. In fact, it is very difficult for the Li^+^ absorption on C atoms, but C–F semi-ionic bond can enhance the Li^+^ adsorption capacity of C atoms, enabling higher capacity for energy storage [39]. The strong interaction between the functional groups on COF_BTMB-TP_ skeleton and Li^+^ is supported by the distribution of Mulliken bonds. The exceedingly low lattice energy of C=O (‒0.454 eV), C–N (‒0.555 eV) and C–F (‒0.301 eV) bonds make it easier for them to compete with PF_6_^−^ and obtain more Li^+^. In addition, the calculated electrostatic potential distribution of COF_BTMB-TP_ (inset in Fig. 2g) shows that the electrons in COF_BTMB-TP_ skeleton can be transferred into C–F (red area), leading to high electron cloud density in C–F region. Therefore, the Li^+^ migration process from electrolyte components to COF_BTMB-TP_ is drawn in Fig. 2h as follows: the lithium-ions undergo the desolvation from electrolyte component due to the strong electronegative C–F, subsequently absorbing onto the COF_BTMB-TP_, simultaneously affected by repulsive force of C–F on electrolyte anions.

### Investigation of Li^+^ Diffusion Rate and Capacity Matching

**Fig. 3:**
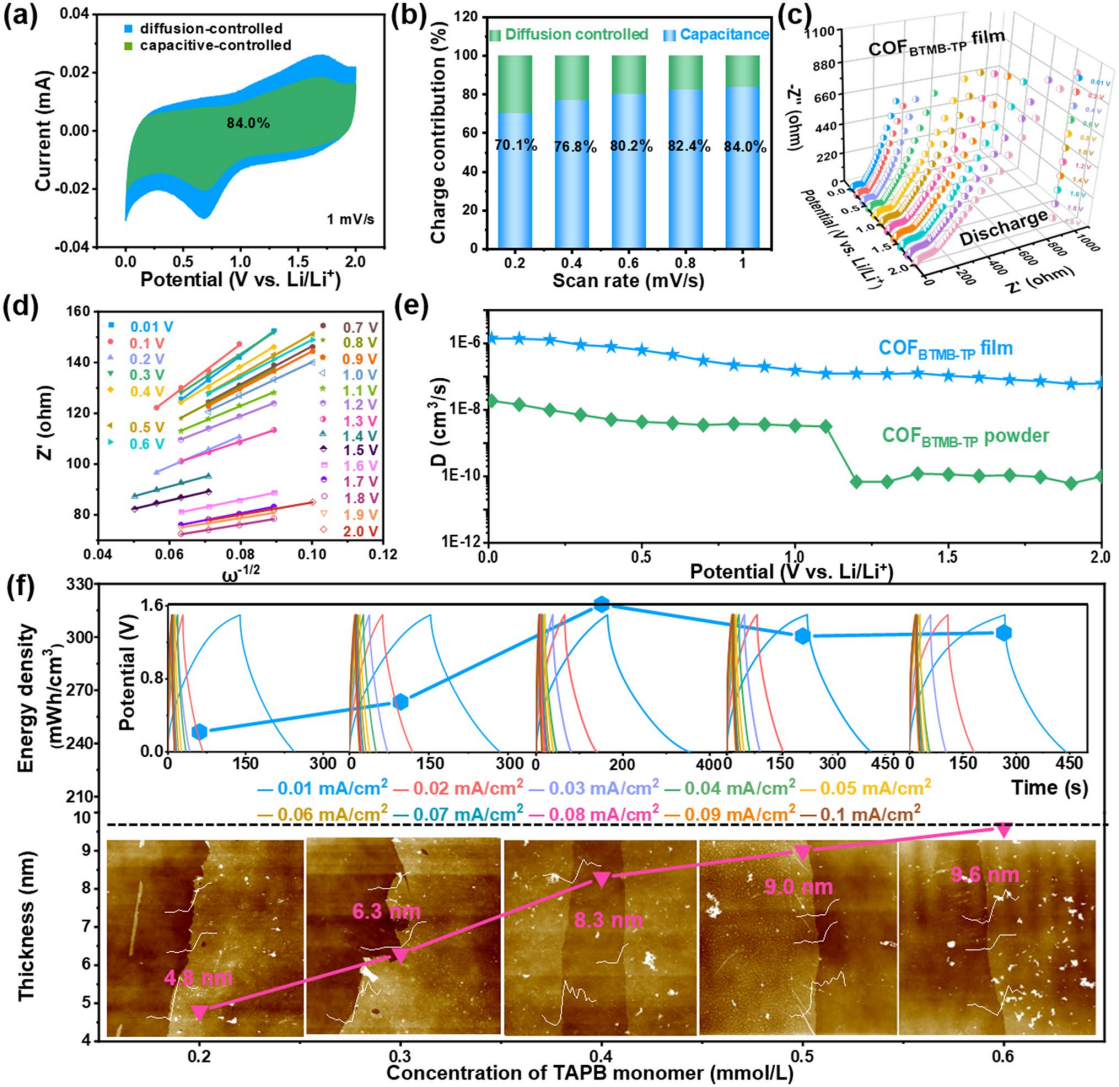
Capacitive contribution marked CV curve at 1 mV s^−1^ (**a**), surface capacitance and diffusion-controlled proportion (**b**), dynamic Nyquist plots at various discharge potentials (**c**), plots of Z’ *vs.* ω^−1/2^ (**d**) of COF_BTMB-TP_ nanofilms; Li^+^ diffusion coefficient of COF_BTMB-TP_ nanofilms and powder (**e**); effect of the concentration of TAPB monomer on the thickness of COF_TAPB-BPY_ nanofilm and energy density of COF_TAPB-BPY_//COF_BTMB-TP_ nanofilm LIC device (**f**)

In order to figure out the internal kinetics of COF_BTMB-TP_ nanofilm in energy storage process, the surface capacitance and diffusion-controlled contribution for COF_BTMB-TP_ electrode were analyzed at a series of scan rates. In Fig. 3a, the green-highlighted surface capacitance contribution constitutes a significant portion of CV integral area, amounting to 84% at a scan rate of 1 mV s^−1^ and this contribution increases with the scan rates escalate. The proportion of surface capacitance contribution is high, reaching 70.1%, 76.8%, 80.2%, 82.4% and 84.0% at scan rate of 0.2, 0.4, 0.6, 0.8 and 1.0 mV s^−1^, respectively (Fig. 3b). This phenomenon explains the excellent charge storage kinetics behavior of COF_BTMB-TP_ electrode, which perfectly match the capacitive kinetic process of the LIC cathode. This kinetic process can be also effectively evaluated by ion diffusion coefficient from EIS measurement. In Fig. 3c, dynamic EIS spectra of COF_BTMB-TP_ nanofilm at different discharge potentials exhibits the semicircular arc and straight line at high and low frequent region, respectively; these features correspond to the charge transfer resistance and ion diffusion *Warburg* resistance. At the ion diffusion region, these plots of Z’ versus *ω*^−1/2^ (Fig. 3d) were summarized to calculate the slope values at different potentials (Fig. S5e), which were subsequently employed to calculate the Li^+^ diffusion coefficient based on Eq. (6). Similarly, as a comparison, the calculated Li^+^ diffusion coefficient of bulk COF_BTMB-TP_ can reach 5.6 × 10^−11^ cm^2^ s^−1^ (Fig. S5f), surpassing many other electrode materials such as LiNi_0.5_Mn_0.5_O_2_ (3.7 × 10^−13^ cm^2^ s^−1^) [40] and LFP/CZIF-8 (1.17 × 10^−13^ cm^2^ s^−1^) [41]. In view of the nano-level thickness of film, the volumetric Li^+^ diffusion coefficient at different potentials (Fig. 3e) show that COF_BTMB-TP_ nanofilm exhibits the significantly higher volumetric Li^+^ diffusion coefficient of 1.15 × 10^−6^ cm^3^ s^−1^, compared with that of bulk COF_BTMB-TP_ (1.86 × 10^−8^ cm^3^ s^−1^). Moreover, with the increasing potential, the attenuation of Li^+^ diffusion coefficient appears at 1.2 V versus Li/Li^+^ for bulk COF_BTMB-TP_; there is no change for COF_BTMB-TP_ nanofilm. This suggests the wider operating voltage range for highly ordered COF_BTMB-TP_ nanofilm. To bridge the enormous gap of cathode/anode in the output capacity arising from different energy storage mechanisms in LICs, the thickness values of cathodic COF_TAPB-BPY_ are adjusted about 4.8, 6.3, 8.3, 9.0, and 9.6 nm (Fig. 3f), by varying the concentration of adding TAPB at 0.2, 0.3, 0.4, 0.5, 0.6 mmol L^−1^ and corresponding BPY monomers (molar ratio of BPY to TAPB = 1.5) to ensure a balanced charge of Q_+_  = Q_−_. It can be found that the thickness of cathodic COF_TAPB-BPY_ is not increased significantly after adding 0.3 mmol L^−1^ TAPB and 0.45 mmol/L BPY monomers. Correspondingly, the energy density of assembled LIC devices incorporated these cathodic COF_TAPB-BPY_ nanofilms shows the similar trend with the thickness of cathodic nanofilms. Therefore, the COF_TAPB-BPY_ nanofilm with a thickness of 8.3 nm was selected to serve as cathode in the LIC, matched with the COF_BTMB-TP_ nanofilm anode to ensure capacity balance. In the inset of Fig. 3f, the coulomb efficiency of the assembled LIC device decreases to a certain extent at the current density of 0.01 mA cm^−2^, which can be explained by the impact of internal resistance from incomplete electrode activation at the low current density.

### Electrochemical Performance of COF_TAPB__-BPY_//COF_BTMB__-TP_ Nanofilm LIC

**Fig. 4:**
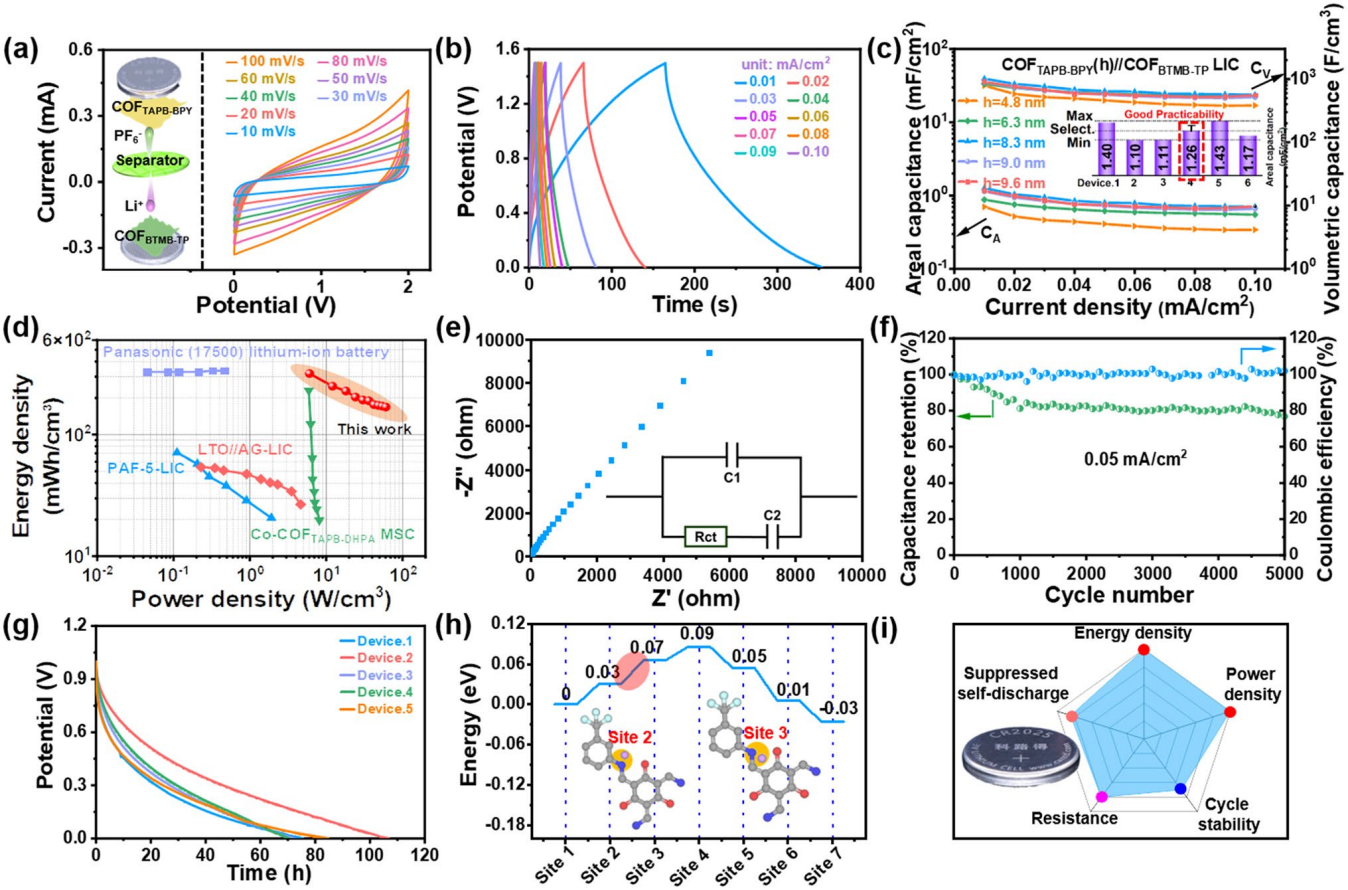
CV (**a**) and GCD (**b**) curves, *C*_A_ and *C*_V_ (**c**), *Ragone* (**d**) and *Nyquist* (**e**) plots, cycle stability and coulombic efficiency (**f**), self-discharge performance (**g**) of the COF_TAPB-BPY_//COF_BTMB-TP_ nanofilm LIC; energy barrier of Li^+^ diffusion control step in anode COF_BTMB-TP_ structure (**h**); performance evaluation of COF_TAPB-BPY_//COF_BTMB-TP_ nanofilm LICs (**i**)

Since above kinetics and capacity matching, the pre-lithiated COF_BTMB-TP_ and optimal COF_TAPB-BPY_ nanofilm with the thickness of 8.3 nm were integrated into a COF_TAPB-BPY_//COF_BTMB-TP_ nanofilm LIC device as the cathode/anode, respectively, as shown in the internal structure diagram of button-type device (inset in Fig. 4a). The COF_TAPB-BPY_//COF_BTMB-TP_ nanofilm LIC device demonstrates the excellent capacitive performance, exhibited by the standard shuttle-like shaped CV curve (Fig. 4a) and quasi-straight GCD curves without obvious platforms (Fig. 4b). In details, both the areal and volumetric specific capacitances of LIC devices increase continuously with the thickness of cathodic COF_TAPB-BPY_ nanofilm rises from 4.8 to 8.3 nm. And then they remain constant even with the use of an 9.0 or 9.6 nm COF_TAPB-BPY_ nanofilm cathode and COF_BTMB-TP_ anode (Figs. 4c and 3g). All these involved data have been also evaluated by reproducibility test including 6 identical devices (inset in Fig. 4c), exhibiting the reasonable error margin, which can prove the robust feasibility of COF_TAPB-BPY_//COF_BTMB-TP_ nanofilm LIC device. Figure 4d collects the *Ragone* plots of energy and power density for this COF_TAPB-BPY_//COF_BTMB-TP_ nanofilm LIC device (318 mWh cm^−3^ at 6 W cm^−3^), together with reported film-type LICs and supercapacitors as the contrast. The COF_TAPB-BPY_//COF_BTMB-TP_ nanofilm LIC device outperforms reported LICs in highest energy/power density, including PAF-5-LIMC (71.1 mWh cm^−3^ at 1.9 W cm^−3^) [42], LTO//AG-LIMCs-80 (53.5 mWh cm^−3^) [43], as well as supercapacitors such as Co-COF_TAPB-DHPA_ (230.4 mWh cm^−3^ at 5.9 W cm^−3^) [19]. Notably, this COF_TAPB-BPY_//COF_BTMB-TP_ nanofilm LIC device achieves a comparable energy density to that of the commercial Panasonic (17,500) lithium-ion battery, which is about 340 mWh cm^−3^. This similarity is particularly pronounced at higher power densities. The *Nyquist* plots (Fig. 4e) of COF_TAPB-BPY_//COF_BTMB-TP_ nanofilm LIC shows the negligible semicircular at high frequency region, indicating the low charge transfer resistance and interface impedance. At high frequency region, the linear portion with a high slope can be fitted by the equivalent circuit to obtain the low Li^+^ diffusion resistance of about 2.1 Ω. In addition, this COF_TAPB-BPY_//COF_BTMB-TP_ nanofilm LIC device exhibits acceptable long-cycle stability, with 77% capacitance retention after 5000 cycles at 0.05 mA/cm^2^ (Fig. 4f) and coulombic efficiency of about 100%. The common self-discharge phenomenon of COF_TAPB-BPY_//COF_BTMB-TP_ nanofilm LIC device was tested by collecting the potential signal after 10 charge/discharge cycles and standing for 1 h at 1 V. In Fig. 4g, the COF_TAPB-BPY_//COF_BTMB-TP_ nanofilm LIC shows slow self-discharge with up to 80 h self-discharge time in most parallel experiments. This phenomenon is primarily attributed to the presence of an energy barrier of 0.04 eV within the anode COF_BTMB-TP_ structure, which controls the Li^+^ diffusion rate (Fig. 4h), as reported in literature[44]. Upon comparison, the surprising electrochemical performances of COF_TAPB-BPY_//COF_BTMB-TP_ nanofilm LIC device can be evaluated in the current level, such as the extremely high energy and power density, good cycle stability, slow self-discharge, and low resistance (Fig. 4i).

## Conclusion

In summary, two free-standing COF nanofilms were synthesized by the reversible Schiff base polymerization on the SDBS surfactant derived gas–liquid interface. The COF_BTMB-TP_ nanofilm, featuring–CF_3_, C=O, and C–N groups, was assembled into an all-COF nanofilm-structured LIC as the anode and COF_TAPB-BPY_ nanofilm with inherent skeleton nitrogen atoms as the cathode. The strong electronegative–CF_3_ groups can adjust the partial electron cloud density for Li^+^ migration, ensuring the rapid kinetic process of anodic COF_BTMB-TP_ nanofilm, to match the capacitance-type cathodic COF_TAPB-BPY_ nanofilm. The cathodic COF_TAPB-BPY_ nanofilm with the thickness of 8.3 nm can fit the anodic COF nanofilm in the capacity. On basis of both kinetics and capacity balance, the COF_TAPB-BPY_//COF_BTMB-TP_ nanofilm LIC can exhibit the high volumetric energy density of 318 mWh cm^−3^ at 6 W cm^−3^, long-cycle stability (77% after 5000 cycles), slow self-discharge, and low resistance. This work provides a new idea for the design of high-performance film-type LIC devices.

## Supplementary Information

Below is the link to the electronic supplementary material.

Supplementary file1 (PDF 1537 KB)
