# Supplementary material for: All-Covalent Organic Framework Nanofilms Assembled Lithium-Ion Capacitor to Solve the Imbalanced Charge Storage Kinetics
Source: Nanomicro Lett. 2024 Feb 15;16:116. doi: 10.1007/s40820-024-01343-2 (PMC10869674; doi:10.1007/s40820-024-01343-2)
Supplement: Supplementary file 1 — Supplementary file1 (PDF 1537 KB) [file 40820_2024_1343_MOESM1_ESM.pdf]

Supporting Information for

All-Covalent Organic Frameworks Nanofilms Assembled Lithium-Ion Capacitor to Solve the Imbalanced Charge Storage Kinetics

Xiaoyang Xu[1, ‡], Jia Zhang[1,2,‡], Zihao Zhang[1], Guandan Lu[1], Wei Cao[1], Ning Wang[2], Yunmeng Xia[1, 2], Qingliang Feng[2, *], Shanlin Qiao[1, 3, *]

[1]College of Chemistry and Pharmaceutical Engineering, Hebei University of Science and Technology, Shijiazhuang 050018, People's Republic of China

[2]School of Chemistry and Chemical Engineering, Northwestern Polytechnical University, Xi'an 710072, People's Republic of China

[3]Hebei Engineering Research Center of Organic Solid Photoelectric Materials for electronic information, Shijiazhuang 050018, People's Republic of China

‡Xiaoyang Xu and Jia Zhang contributed equally to this work.

*Corresponding authors. E-mail: ccpeslqiao@hebust.edu.cn (S. Qiao); fengql@nwpu.edu.cn (Q. Feng)

Supplementary Figures and Tables

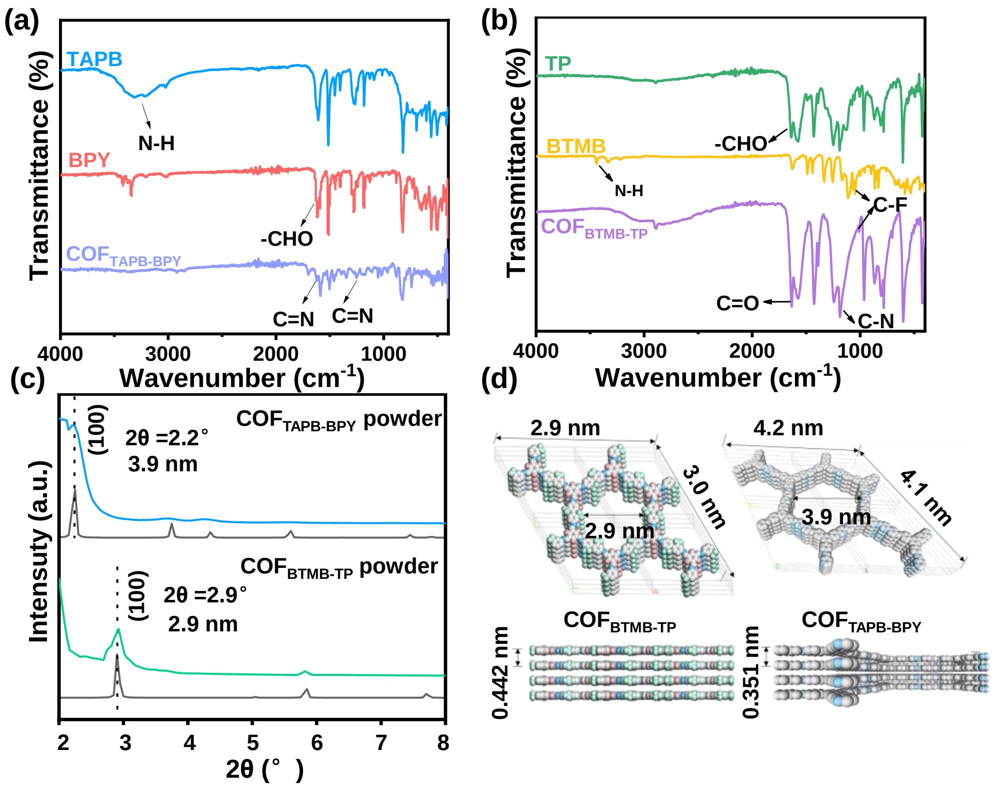

Fig. S1. FT-IR spectra (**a-b**), XRD (**c**) and MS calculation result (**d**) of $COF_{TAPB-BPY}$

and $COF_{BTMB-TP}$

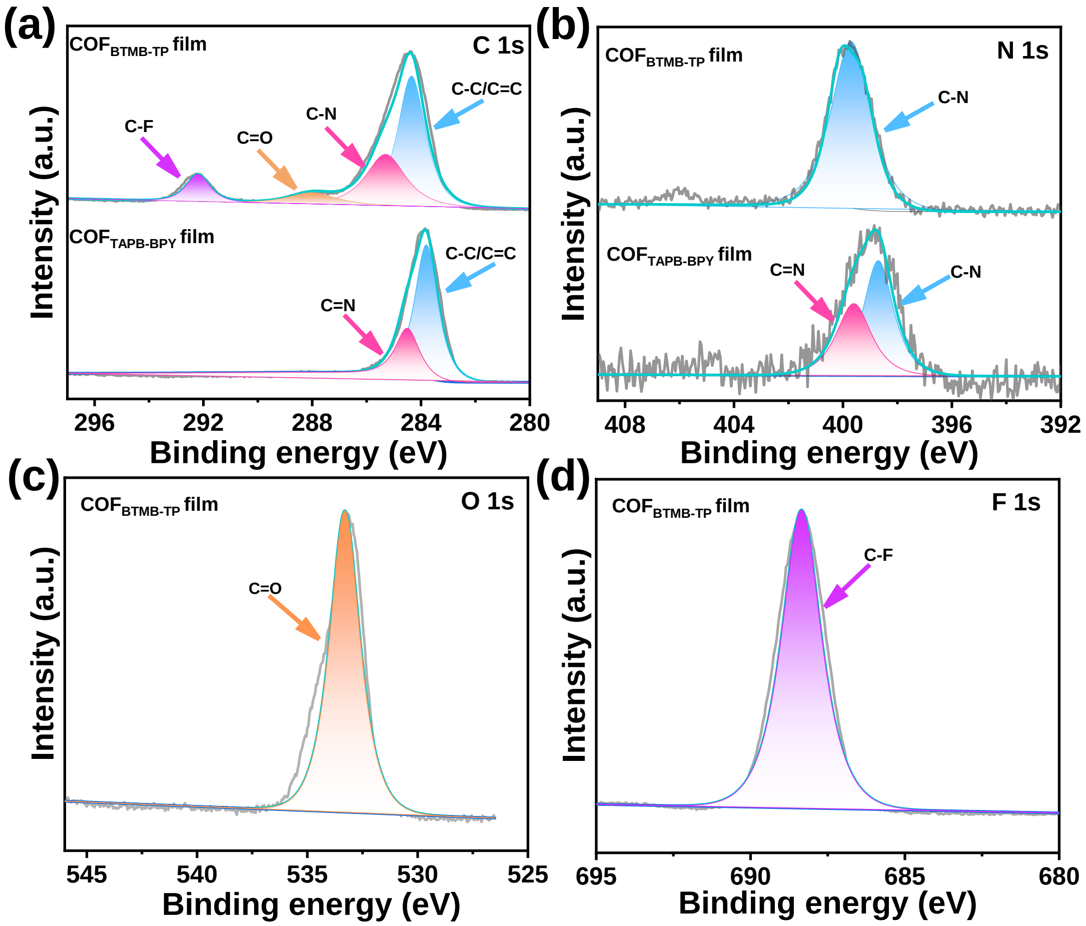

Fig. S2 C 1s (**a**), and N 1s (**b**) core-level spectra of $COF_{TAPB-BPY}$ and $COF_{BTMB-TP}$ film; O 1s (**c**), and F 1s (**d**) of $COF_{BTMB-TP}$ film

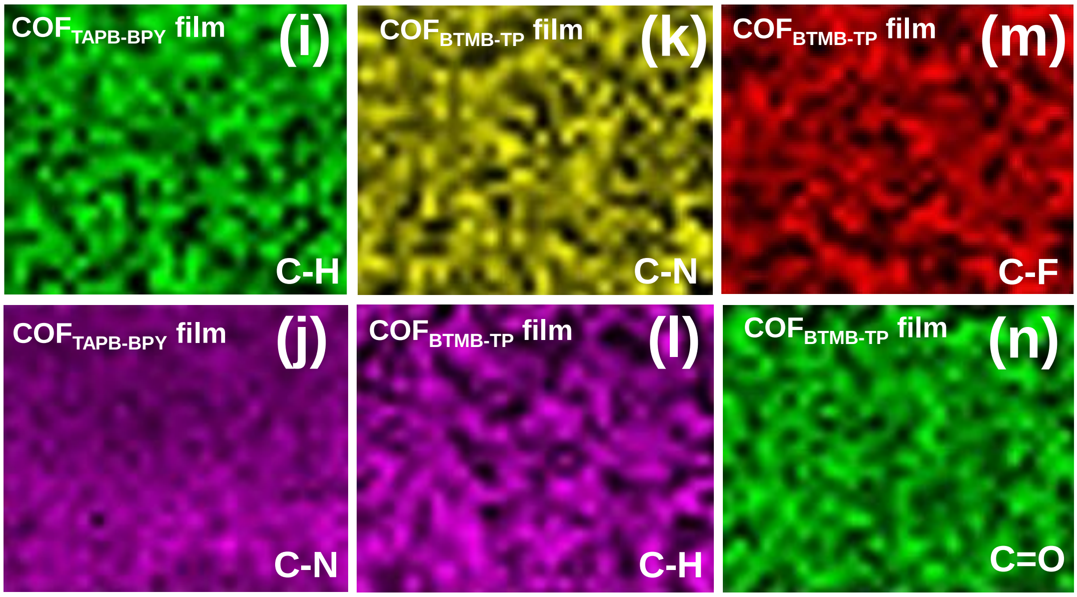

Fig. S3 Raman mapping (a-f) of $COF_{TAPB-BPY}$ and $COF_{BTMB-TP}$ nanofilms

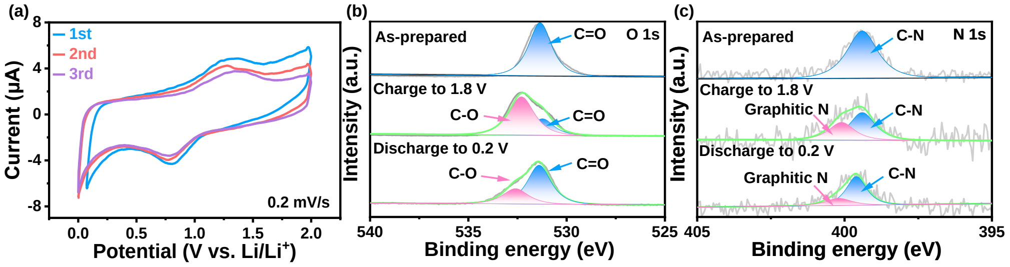

Fig. S4 CV curves at 0.2 mV/s in the first 3 cycles; Ex-situ XPS spectra of in $LiClO_4$ (1 M, in EC:PC=1:1 Vol%) alternative electrolyte

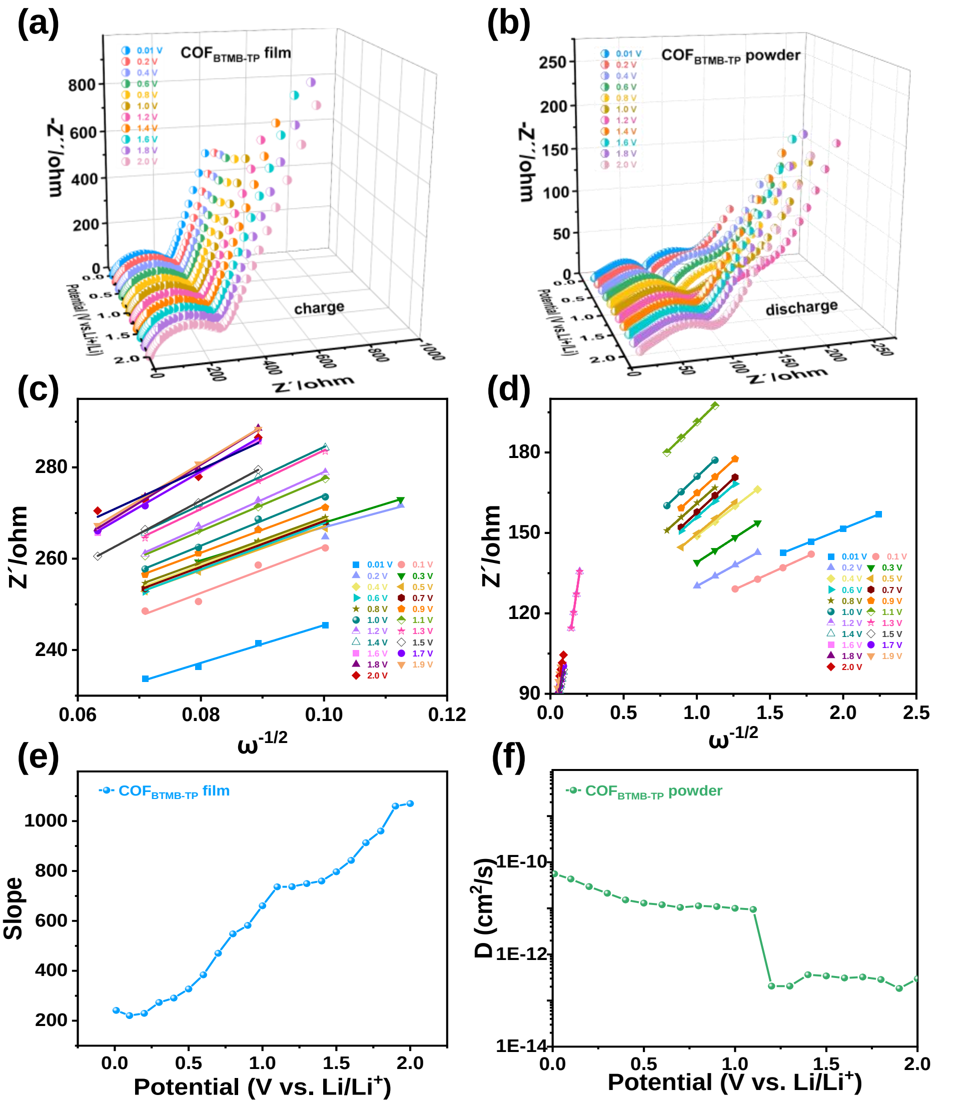

Fig. S5 Nyquist plots of $COF_{BTMB-TP}$ nanofilms at various potential during charge processes (**a**) and $COF_{BTMB-TP}$ powder during discharge processes (**b**), *Z'* as a function of the $\omega^{1/2}$ plot in the low frequency range of $COF_{BTMB-TP}$ nanofilms charged (**c**) and $COF_{BTMB-TP}$ powder discharged (**d**) (the slope of fitting curves is the Warburg factor,

σ_W), the slope values of $COF_{BTMB\text{-}TP}$ nanofilms discharged (**e**) and $COF_{BTMB\text{-}TP}$ powder discharged (**f**) at different potentials

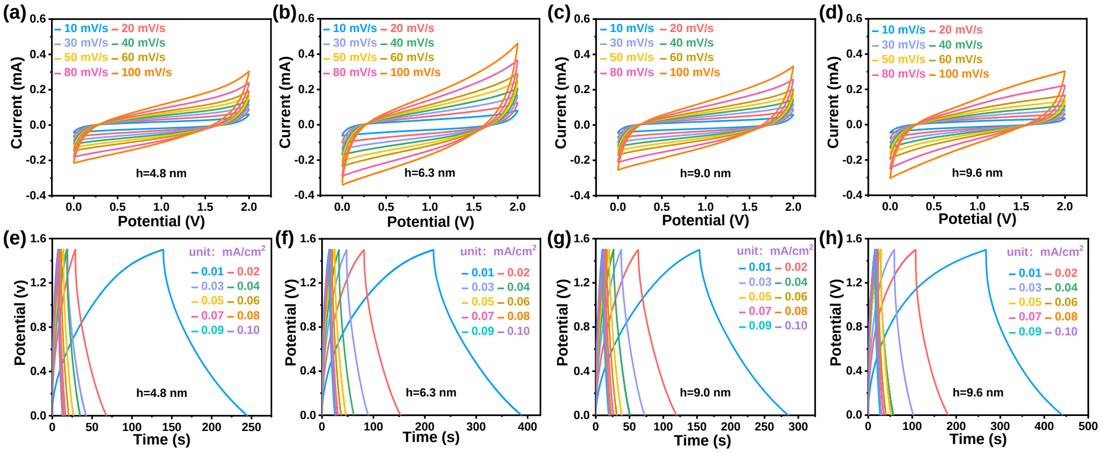

Fig. S6 CV (a-d) and GCD (e-h) curves of $COF_{TAPB\text{-}BPY}$ (*h*)//$COF_{BTMB\text{-}TP}$ LIC devices (*h* is the thickness of $COF_{TAPB\text{-}BPY}$ nanofilms, *h* = 4.8, 6.3, 9.0, and 9.6 nm)
